# Screening of hepatocyte proteins binding with C-terminally truncated surface antigen middle protein of hepatitis B virus (MHBst^167^) by a yeast two-hybrid system

**DOI:** 10.3892/mmr.2014.2356

**Published:** 2014-06-26

**Authors:** ZHI QUN LI, ENQIANG LINGHU, WAN JUN, JUN CHENG

**Affiliations:** 1 Department of Gastroenterology and Hepatology, Chinese 261 General Hospital, Beijing 100853, P.R. China; 2 Department of Gastroenterology and Hepatology, Chinese PLA General Hospital, Beijing 100853, P.R. China; 3 Institute of Infectious Diseases, Ditan Hospital, Capital Medical University, Beijing 100015, P.R. China

**Keywords:** hepatitis B virus, MHBst^167^, yeast two-hybrid

## Abstract

The function of middle hepatitis B surface protein C-terminally truncated at amino acid position 167 (MHBst^167^) is not currently clear. This study aimed to screen and identify the proteins that interact with MHBst^167^ in hepatocytes using a yeast two-hybrid system, and to explore the effects of MHBst^167^ in the development of hepatocellular carcinoma and precancerous diseases of the liver. The MHBst^167^ gene was amplified by polymerase chain reaction (PCR) and cloned into a pGEM-T vector. The target region was sequenced and the constructed bait plasmid, pGBKT7-MHBst^167^, was transformed into AH109 yeast cells. The transformed AH109 cells were then mated with Y187 yeast cells containing the fetal liver cDNA library plasmid using a yeast two-hybrid system. The false positives were eliminated and the true positive clones were selected by PCR and sequencing analysis. The pGBKT7-MHBst^167^ bait plasmid was successfully constructed and 66 clones grew in the selective synthetic defined media lacking leucine, tryptophan, histidine and adenine. Fifty-two clones were identified following X-α-Gal selection and segregation analysis. Seven proteins were found to be expressed that could interact with MHBst^167^ in hepatocytes by the yeast two-hybrid system. These results have provided novel insights into the biological functions of MHBst^167^.

## Introduction

Previous studies have indicated that numerous proteins are associated with the development of hepatocellular carcinoma (HCC) (1–8). Hepatitis B virus (HBV) encodes three envelope proteins in the pre-S/S open reading frame that are termed large, middle and small (major) surface proteins (9). Following the integration of viral sequences into the genome, the translation of C-terminally truncated surface proteins of HBV frequently occurs. It has been suggested that the expression of certain genes, activated by these translated viral sequences, may contribute to hepatocarcinogenesis (10).

In a previous study (10), the transactivating potential of middle hepatitis B surface protein C-terminally truncated at amino acid 167 (MHBst^167^) on the HBV regulatory element was investigated. The data indicated that MHBst^167^ is a pleiotropic, non-liver-specific transactivator which can modulate ubiquitous transcription factors that are associated with proliferation and inflammation (10). In order to further reveal the biological roles of MHBst^167^, the present study investigated cellular proteins interacting with the carboxyl terminus of MHBst^167^, an important functional region. To identify these proteins, a GAL4-based yeast two-hybrid system (Clontech Laboratories, Inc., Mountain View, CA, USA), using the MHBst^167^ cDNA as bait, was utilized to screen a human fetal liver cDNA library. The aim of the study was to provide novel insights into the biological functions of MHBst^167^.

## Materials and methods

### Yeast strains and plasmids

The Matchmaker^®^ GAL4-based two-hybrid system 3 and vector pACT2 containing a human fetal liver cDNA library were obtained from Clontech Laboratories, Inc. The yeast strain AH109 (MATα, trp1–901, leu2–3, 112, ura3–52, his3–200, gal4Δ, gal80Δ, LYS2:GAL1_UAS_-GAL1_TATA_-HIS3, GAL2_UAS_-GAL2_TATA_-ADE2, URA3:MEL1_UAS_-MEL1_TATA_-LacZ) contained the pGBKT7–53 plasmid, encoding the DNA-binding domain (DNA-BD) mouse p53 fusion protein. The yeast strain Y187 (MATα, ura3–52, his3–200, ade2–101, trp1–901, leu2–3, 112, gal4, gal80, met-, URA3::GAL1_UAS_-GAL1_TATA_-lacZ, MEL1) contained the pTD1-1 plasmid, in which vector pACT2 encoded the AD/simian virus 40 large T antigen fusing protein. AH109 was used for cloning bait plasmids and Y187 was used for cloning library plasmids. The yeast-*Escherichia coli* shuttle plasmids pGBKT7 DNA-BD cloning plasmid, pGADT7 DNA-activation domain (DNA-AD) cloning plasmid, pGBKT7–53 control plasmid, pGADT7, pGBKT7-Lam control plasmid and pCL1 were obtained from Clontech Laboratories, Inc.

### Chemical agents and culture media

The pGEM-T vector and *Taq* DNA polymerase were purchased from Promega Corp. (Madison, WI, USA). T4 DNA ligase, *Eco*RI and *Bam*HI restriction endonucleases were purchased from Takara (Shiga, Japan), and c-Myc monoclonal antibody and goat anti-mouse immunoglobulin G antibody conjugated with horseradish peroxidase were obtained from Zhongshan Company (Zhongshan, China). Tryptone and yeast extracts were obtained from Oxoid (Thermo Fisher Scientific, Inc., Waltham, MA, USA). X-α-Gal and yeast peptone dextrose adenine, synthetic defined (SD)/-tryptophan (Trp), SD/-leucine (Leu), SD/-Trp/-Leu, SD/-Trp/-Leu/-histidine (His) and SD/-Trp/-Leu/-His/-adenine (Ade) media were purchased from Clontech Laboratories, Inc. Glass beads were purchased from Sigma (St. Louis, MO, USA), while lysis buffer [20 g/l Triton X-100, 10 g/l SDS, 10 mmol/l NaCl, 10 mmol/l Tris-HCl (pH 8.0) and 1 mmol/l Na_2_EDTA] and phenol, chloroform and isoamyl alcohol (volume fraction 25:24:1) were also obtained from Clontech Laboratories, Inc.

### Construction of bait plasmid and expression of MHBst^167^

**Figure 1:**
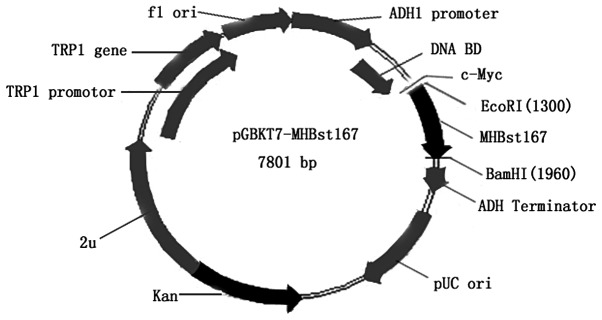
Structure of the bait plasmid pGBKT7- MHBst^167^.

The extracted MHBst^167^ DNA was amplified by polymerase chain reaction (PCR) from the A7 plasmid (HBV strain subtype adr). The primer sequences, which contained *Eco*RI and *Bam*HI sites, respectively, for cloning were as follows: Sense, 5′-gaattcatggtcaccttgaggtgg-3′; antisense, 5′-ggatcccaaacaggagatgaaggtcct-3′. The PCR conditions were as follows: 94°C for 4 min, denaturation at 94°C for 50 sec, annealing at 58°C for 50 sec, extension at 72°C for 1 min, 35 cycles. The PCR product was cloned into the pGEM-T vector and the primary structure of the insert was confirmed by direct sequencing. The auto-sequencing assay was performed by the Shanghai Hua Nuo Biological Corporation (Shanghai, China). The fragment encoding the MHBst^167^ was digested from the pGEM-T-MHBst^167^ using *Eco*RI and *Bam*HI restriction enzymes, and ligated into the pGBKT7 vector. The pGBKT7 vector expressed proteins fused to amino acids 1–147 of the GAL4 DNA-BD and the pGADT7 vector expressed proteins fused to amino acids 768–881 of the GAL4 DNA-AD. The MHBst^167^ gene was inserted into the pGBKT7 multiple cloning site. The resulting plasmid, pGBKT7-MHBst^167^ (Fig. 1), containing the full-length MHBst^167^ gene, could direct expression of the DNA-BD, c-Myc and MHBst^167^ fusion protein. The plasmid was transformed into yeast strain AH109 using a lithium acetate method as previously described (11).

### Yeast two-hybrid screen

The yeast two-hybrid screening procedure used was a modification of the method described by Gietz *et al* (11). The pACT2-cDNA plasmid genome was isolated following the standard protocol. AH109 yeast cells containing pGBKT7-MHBst^167^ were transformed into the Y187 yeast strain containing the pACT2-cDNA liver library (Clontech Laboratories, Inc.) by the lithium acetate method, in accordance with the manufacturer’s instructions. Cells were plated and selected for on quadruple dropout (QDO) media lacking leucine, tryptophan, histidine and adenine. After 6–18 days of growth at 30°C, the yeast colonies were transferred onto plates containing X-α-Gal to check for the expression of the MEL1 reporter gene. Positive interactions were detected by the appearance of blue-colored colonies. Segregation analysis and mating experiments were performed to exclude the false positives, ensuring that only true positive colonies were selected. Following the sequencing of the positive colonies, the sequences were BLASTed with GenBank (blast.ncbi.nlm.nih.gov/Blast.cgi) to analyze the function of the genes.

## Results

### Identification of the plasmid

**Figure 2:**
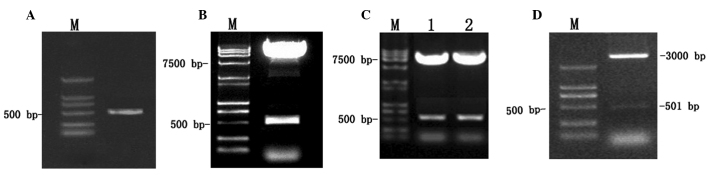
Construction and identification of plasmids by agarose gel electrophoresis. (A) Polymerase chain reaction product MHBst^167^ (501 bp). (B) Restriction enzyme digestion of pGBKT7-MHBst^167^ plasmids by *Eco*RI/*Bam*HI (pGBKT7, 7,300 bp; MHBst^167^, 501 bp). (C) Restriction enzyme digestion of (Lane 1) pGBKT7-MHBst^167^ and (Lane 2) pGADT7-MHBst^167^ by *Eco*RI/*Bam*HI. (D) Restriction enzyme digestion of pGEM-T-MHBst^167^ plasmids by *Eco*RI/*Bam*HI. M, DL2000 DNA marker; bp, base pairs.

**Figure 3:**
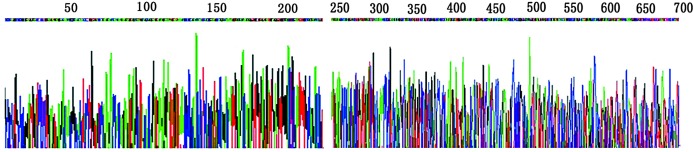
Sequence of the pGEM-T-MHBst^167^ by direct sequencing.

The 501-bp fragment of MHBst^167^ was generated by PCR amplification of the HBV plasmid (subtype adr), sequenced and analyzed using Vector NTI^®^ 6 (Life Technologies, Carlsbad, CA, USA) and BLAST database homology. The PCR product was cloned with the pGEM-T vector. Following digestion with *Eco*RI/*Bam*HI restriction enzymes, the fragments were ligated in-frame into the pGBKT7 and pGADT7 *Eco*RI/*Bam*HI sites. Restriction enzyme analysis of pGBKT7-MHBst^167^ and pGADT7-MHBst^167^, respectively, with *Eco*RI/*Bam*HI yielded two products: 7,300 bp empty pGBKT7 and 501 bp MHBst^167^, and 7,900 bp empty pGADT7 and 501 bp MHBst^167^. Analysis of the PCR products by agarose gel electrophoresis (Fig. 2) showed the products with the expected size (501 bp, MHBst^167^). The pGEM-T-MHBst^167^ sequence was confirmed by direct sequencing (Fig. 3).

### Screening of the fetal liver cell cDNA library

**Figure 4:**
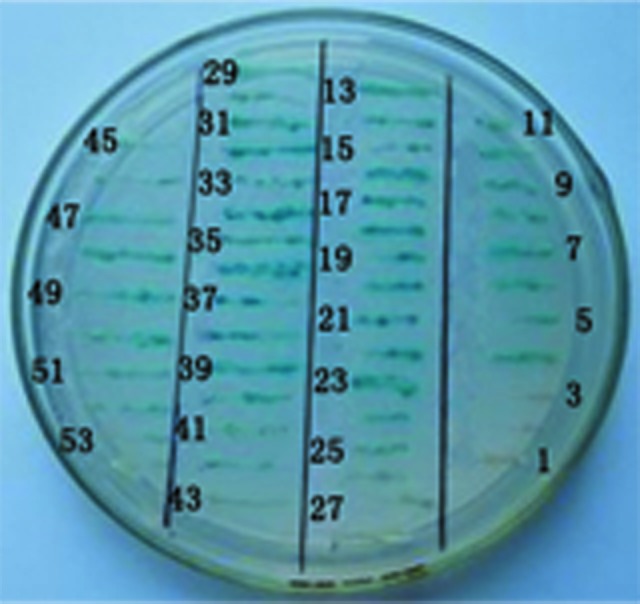
Screening of blue colonies containing only the MEL1 reporter gene on the X-α-Gal medium lacking leucine, tryptophan, histidine and adenine.

**Figure 5:**
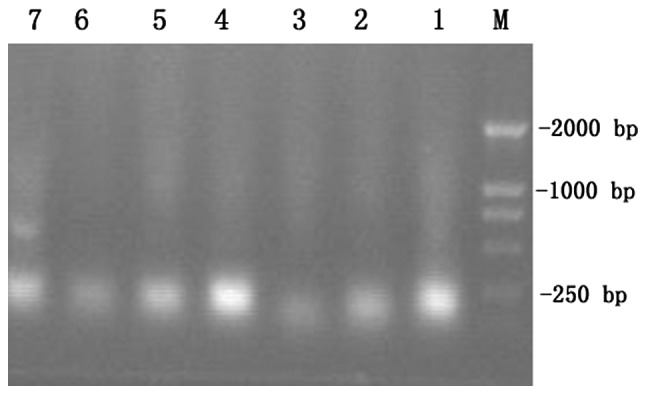
Identification of different colonies with *Bgl*II digestion. M, DL2000 DNA marker; bp, base pairs.

Plasmids from the blue-colored colonies containing only pGBKT7-MHBst^167^, as the bait for screening the human fetal liver cell cDNA library, were isolated. Sixty-six clones grew on the QDO media. The clones were further selected for by X-α-Gal assay and, again, blue colonies were picked. Following elimination, 52 positive clones were further tested by the specificity of the selective SD/-Trp-Leu-His-Ade/X-α-Gal media and expression (Fig. 4). Since pACT2-cDNA plasmids contained two restriction endonuclease sites for recognition by *Bgl*II either side of the multiple cloning site, the gene fragments of the fetal liver cell cDNA library (pACT2-cDNA) screened were released by *Bgl*II digestion (Fig. 5).

### Analysis of cDNA sequencing and homology

Seven positive colonies grew on the selective SD/-Trp-Leu-His-Ade/X-α-Gal media. These colonies were prescreened by *Bgl*II digestion to ensure that only colonies with different inserts were subjected to sequencing. The seven colonies from the cDNA library were sequenced and analyzed using BLAST software provided by the National Center for Biotechnology Information. The seven sequences had high similarity to known genes. A summary of the identified genes is shown in Table I.

## Discussion

Genetic alterations associated with human HCC have been reported for numerous genes; however, at present these are not sufficient to distinguish between HCC and precancerous liver diseases, including hepatitis, hepatic fibrosis and cirrhosis (12). It has previously been suggested that the expression of certain genes, activated by translated HBV sequences, may contribute to hepatocarcinogenesis (10). Large HBV surface protein and MHBst belong to the HBV PreS2 activator protein family. PreS2 activators have been suggested to act in a similar manner to promoters of tumorigenesis, modulating the control of proliferation through the activation of certain key enzymes (13). The ability of PreS2 activator proteins to activate transcription is associated with the cytoplasmic orientation of the PreS2 domain. While MHBst represents a standard for PreS2 activator proteins, the full-length MHBs exhibits no transcriptional activator activity, since the PreS2 domain is oriented into the lumen of the endoplasmic reticulum. For functional MHBst to be generated from the full-length MHBs, the 3′ end of the preS2/S gene, which encodes the last 70 amino acids in the sequence, must be deleted during the integration process. This sequence of amino acids corresponds to the third hydrophobic region of the S domain of the protein (14).

The potential of MHBst^167^ to activate transcription is mediated via various ubiquitous transcription factors, which are involved in the activation of proto-oncogenes or genes associated with inflammation. The overexpression of proto-oncogenes induced by MHBst^167^ may be causative of over-proliferation or may maintain mitogenesis; as such, it has been proposed that an association exists between transactivation by integrated HBV sequences and the development of HCC (10).

The present study used a yeast two-hybrid system as an approach for detecting protein-protein interactions. By this method, weak protein interactions may be identified which may not be observed using other *in vitro* assays, such as immunoprecipitation. The yeast two-hybrid system 3 utilized in this study was commercially available from Clontech Laboratories, Inc. (15–17). This system was selected since the promoters modulating HIS3, ADE2 and MEL1 reporter gene expression in the AH109 yeast strain result in significantly fewer false positives. Furthermore, the simple mating protocol is time- and labor-efficient. As such, the system facilitates the identification of rare protein-protein interactions and produces more reproducible results (18,19).

Following the screening of a fetal liver cDNA library, this study identified seven putative clones associated with MHBst^167^. The proteins identified were as follows: i) and ii) *Homo sapiens* ADP-ribosylation factor 1; iii) full-length cDNA clone CS0DM004YC15 of the fetal liver of *Homo sapiens*; iv) *Homo sapiens* ALDOB gene; v) *Homo sapiens* complement component 3 (C3); vi) *Homo sapiens* bacterial artificial chromosome (BAC) clone GS1–306C12 from chromosome 7; and vii) serum spreading factor (SF).

The first interacting protein identified in this study, ADP-ribosylation factor 1, is a GTPase required for the exocytosis of cytotoxic T-lymphocyte antigen 4 (CTLA-4), a process that is also dependent on phospholipase D activity. It has been suggested that ADP-ribosylation factor 1 and phospholipase D act to stimulate the rapid translocation of specialized compartments containing CTLA-4 in regulatory T cells to the plasma membrane (20). Poly(ADP-ribose) polymerase (PARP) is an enzyme predominantly localized in the nucleus and is responsible for catalyzing poly-ADP-ribosylation. The activity of PARP is associated with numerous biological processes, including DNA repair, cell proliferation and malignant transformation. It has been shown that the expression of PARP in HCC is increased in patients with liver cirrhosis, with higher expression levels in less-differentiated tumors (21).

The present study showed that MHBst^167^ also interacted with the full-length cDNA clone CS0DM004YC15 of the fetal liver of *Homo sapiens* and the *Homo sapiens* ALDOB gene. ALDOB is an enzyme involved in glycolysis and gluconeogenesis and is the only isoenzyme of aldolase expressed in differentiated hepatocytes. The impaired function of ALDOB has been associated with hereditary fructose intolerance, a recessively inherited disorder of carbohydrate metabolism. To date, 29 mutations have been identified in the ALDOB gene that impair the functioning of the enzyme (22). ALDOB has additionally been associated with HCC. In a study by Kinoshita *et al* (12), ALDOB was shown to be downregulated in primary HCC tissues in 90% of a cohort of 20 patients, as compared with healthy controls. Therefore, the underexpression of key ALDOB gene products may be important in the development and/or progression of HCC.

C3 is a key protein involved in the complement immune system. In a previous study of patients with chronic liver disease caused by HBV infection, C3 expression levels were revealed to be lower in the serum of patients than those in the serum of healthy controls. Furthermore, the levels of circulating immune complexes (CICs) in the patients were increased (23). It was suggested that the decreased C3 levels may have been a result of decreased synthesis and/or an increased consumption by the CICs (23). Results from a study by Takezaki *et al* (24) indicated that C3 levels may exhibit diagnostic power in the detection of HCC in patients with liver cirrhosis.

The present study showed that MHBst^167^ also interacted with the *Homo sapiens* BAC clone GS1–306C12 from chromosome 7.

Another important protein interacting with MHBst^167^ from the fetal liver cDNA library was serum SF (*Homo sapiens* vitronectin). Human serum SF is a secreted glycoprotein that is involved in the promotion of cell adhesion and spreading. SF has been shown to be synthesized and secreted into culture by HepG2 human HCC cells (25). A study by Inuzuka *et al* (26) analyzed plasma vitronectin levels in patients with liver disease and compared these levels with various parameters of liver function and the severity of liver cirrhosis, graded according to Child’s criteria. The plasma vitronectin level was low in all liver disease groups as compared with that in the healthy controls, although the difference was only significant between the controls and patients with HCC and decompensated cirrhosis. Furthermore, the vitronectin level was inversely correlated with the severity of cirrhosis. These results suggested that the plasma vitronectin level may be used as an indicator of the synthetic function of the liver in patients with liver diseases and that it may also be a marker of the severity of cirrhosis. Immunoelectron microscopy in the same study revealed the presence of vitronectin in the rough endoplasmic reticulum of hepatocytes, as well as around certain cells close to sites of necrosis and on collagen fibers (26).

These interacting proteins, identified by the yeast-two hybrid system, are closely associated with carbohydrate metabolism, tumor development/progression and immunoregulation. These data may provide novel insights into the functions of MHBst^167^, the pathogenesis of HBV-related diseases and malignant transformation. Further studies are required to investigate how the interactions between MHBst^167^ and the proteins identified in this study affect the occurrence and development of chronic hepatitis B, hepatic fibrosis and HCC.

## Figures and Tables

**Table I:** Comparison between positive clones and similar sequences in GenBank.

High similarity to known genes	No. of similar clones	Homology (%)
*Homo sapiens* ADP-ribosylation factor 1, mRNA (cDNA clone IMAGE: 4024984)	2	98
Full-length cDNA clone CS0DM004YC15 of fetal liver of *Homo sapiens*	1	100
*Homo sapiens* ALDOB gene, virtual transcript	1	100
*Homo sapiens* complement component 3, mRNA	1	99
*Homo sapiens* BAC clone GS1–306C12 from 7	1	100
*Homo sapiens* vitronectin (serum spreading factor, somatomedin B, complement S-protein), mRNA	1	98

IMAGE, integrated molecular analysis of genomes and their expression; BAC, bacterial artificial chromosome.

## Abbreviations

HBV: hepatitis B virus
MHBst^167^: middle hepatitis B surface protein C-terminally truncated at amino acid position 167
PCR: polymerase chain reaction
DNA-BD: DNA binding domain
DNA-AD: DNA activation domain
HCC: hepatocellular carcinoma
QDO: quadruple dropout medium lacking leucine, tryptophan, histidine and adenine
CIC: circulating immune complex
BAC: bacterial artificial chromosome
SF: spreading factor
